# Permanent Scatterer InSAR Analysis and Validation in the Gulf of Corinth

**DOI:** 10.3390/s90100046

**Published:** 2009-01-05

**Authors:** Panagiotis Elias, Charalabos Kontoes, Ioannis Papoutsis, Ioannis Kotsis, Aggeliki Marinou, Dimitris Paradissis, Dimitris Sakellariou

**Affiliations:** 1 National Observatory of Athens, Institute of Space Applications and Remote Sensing, Vas. Pavlou and Metaxa str., 15236, Palaia Penteli, Greece. E-Mail: pelias@space.noa.gr; 2 Higher Geodesy Laboratory and Dionyssos Satellite Observatory, National Technical University of Athens, Iroon Polytexneiou 9, 15780, Zografou, Greece. E-Mails: ipapoutsis@space.noa.gr; jkotsis@survey.ntua.gr; amarinou@survey.ntua.gr; dempar@central.ntua.gr; 3 Hellenic Center for Marine Research, Hellinikon, 16604, Athens, Greece. E-Mail: sakell@ncmr.gr

**Keywords:** Ground subsidence, SAR Interferometry, Permanent Scatterer, GPS

## Abstract

The Permanent Scatterers Interferometric SAR technique (PSInSAR) is a method that accurately estimates the near vertical terrain deformation rates, of the order of ∼1 mm year^-1^, overcoming the physical and technical restrictions of classic InSAR. In this paper the method is strengthened by creating a robust processing chain, incorporating PSInSAR analysis together with algorithmic adaptations for Permanent Scatterer Candidates (PSCs) and Permanent Scatterers (PSs) selection. The processing chain, called PerSePHONE, was applied and validated in the geophysically active area of the Gulf of Corinth. The analysis indicated a clear subsidence trend in the north-eastern part of the gulf, with the maximum deformation of ∼2.5 mm year^-1^ occurring in the region north of the Gulf of Alkyonides. The validity of the results was assessed against geophysical/geological and geodetic studies conducted in the area, which include continuous seismic profiling data and GPS height measurements. All these observations converge to the same deformation pattern as the one derived by the PSInSAR technique.

## 1. Introduction

### 1.1. PSInSAR processing

The classic InSAR technique has offered numerous examples for the reliable measurement of ground deformation [1]. The accuracy of this method though, is limited by components relating to spatial and temporal decorrelation, signal delay due to tropospheric and ionospheric disturbances, orbital errors as well as Digital Elevation Model (DEM) artefacts. These components are dealt with the promising Permanent Scatterers Interferometric Synthetic Aperture Radar (PSInSAR) technique [2]. The PSInSAR methodology offers the significant potential of estimating the near-vertical displacement rates with accuracy of the order of 1 mm year^-1^. Thus, this technique is ideal for measuring small-scale ground deformation due to displacements in active fault zones [3, 4], seismic precursor activity and subsidence occurring from manmade construction and drilling activities.

A crucial requirement for this method is the availability of stable targets, which present a dominant reflection component in the radar signal while their scattering characteristics remain unchanged in time. These targets are called Permanent Scatterers (PS) and can be used to remove the above mentioned undesirable components [2]. However, in order to identify a suitable number of PSs in a study area, a large number of SAR acquisitions should be processed. For this purpose, a predefined SAR image is used as master which is combined with the rest of the available SAR image acquisitions to create a set of interferometric calculations. All interferograms are then exploited, including those with large temporal and geometrical baselines.

### 1.2. Gulf of Corinth test site

**Figure 1.**
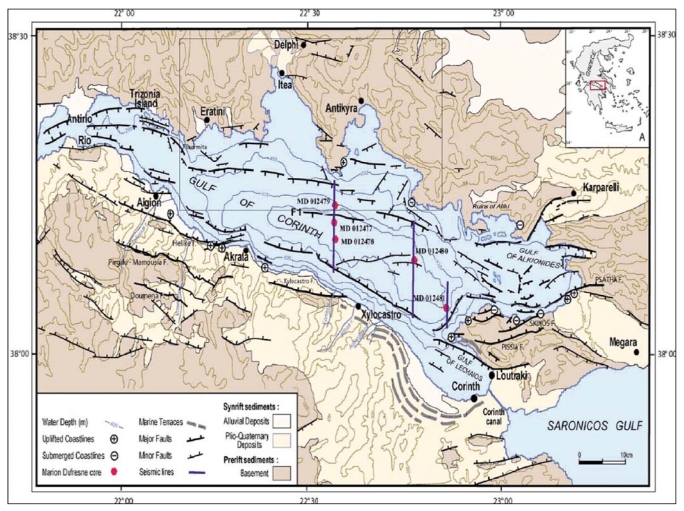
Structural map of the Gulf of Corinth [7] and the location of the test site.

The Gulf of Corinth study area is illustrated in Figure 1. It has been long identified as a site of major importance due to its intense past geophysical activity [5]. It is one of the world's most rapidly extending continental regions and it has one of the highest seismicity rates in the Euro-Mediterranean region, having produced a number of earthquakes with magnitude greater than 5.8: Alkyonides (1981, *M*=6.7), Aigio (1995, *Mw*=6.1), and Galaxidi (1992, *Mw*=5.8). Moreover, the geodetic studies conducted, which were based on GPS observations and InSAR calculations, revealed north – south extension rates across the gulf of up to about 1.5 cm year^-1^ [6] during the last 20 years. The rifting mechanism observed is crucial for the stability of the region as it can lead to submarine slope failures and possible damaging tsunamis. On land, the same fault system causes landslides. However, the aforementioned techniques present limitations for near vertical (∼23° from zenith) movement estimation. This is encountered through PSInSAR processing.

## 2. InSAR analysis

**Figure 2.**
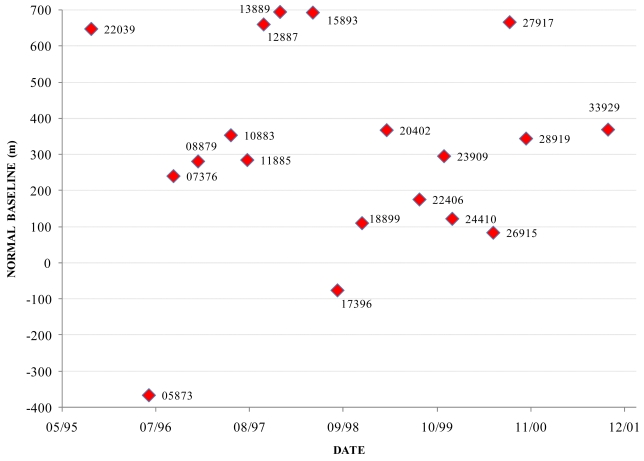
Normal baselines versus the acquisition dates of the scenes. The figure labels correspond to the ERS orbit number of each scene.

The image data used in the present PSInSAR study were acquired from the ERS-1 and ERS-2 satellites, kindly provided by the European Space Agency (ESA). Scene selection was based on three criteria: the first relating to the time span of the scenes, which was selected to be long enough to incorporate a sufficient number of images, but not exceeding a maximum of seven years, in order to avoid temporal decorrelation. The second criterion was the absence of intense non-linear phenomena during the studied period, such as earthquakes, to meet the requirement for linear deformation rates. According to the third criterion, the data set used was characterized by uniform distributions of interferometric baselines and acquisition dates of the scenes (i.e. avoid time gaps, such as the 2002 ERS-2 failure). On the basis of these three criteria a full data set consisted of twenty ERS scenes, with a time span of 6½ years, from June 19^th^, 1995 to October 16^th^, 2001 (Figure 2). The ERS-1 scene acquired on 19^th^ June 1995 (orbit No. 20536) was selected to be the common master scene.

Some necessary pre-processing steps were applied to the raw SAR data. These related to image focusing, image cropping and compensating for zero Doppler centroid. An important step at this stage was the radiometric normalization of the amplitude images in order to achieve enhanced cross-correlation statistics for image registration.

A customized version of the Centre National d'Etudes Spatiales (CNES) DIAPASON software [8] was used to produce the interferometric phases and other necessary by-products, serving as input to the PSInSAR algorithm application. The nineteen interferograms had a cell size of ∼4 m in azimuth and ∼20 m in range and were created from single look images, without applying any pixel averaging techniques. The precise orbital files used were provided by the Delft Institute databases. The DEM of the study area used for interferometric processing was derived by digitizing the 20 m contour lines on existing 1:50000-scale topographic maps. The DEM accuracy was estimated to be of ±10 m. DEM voids were filled with resampled Shuttle Radar Topography Mission (SRTM-3) data [9].

## 3. PSInSAR processing

**Figure 3.**
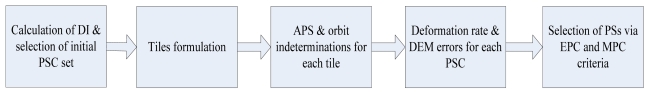
Block diagram illustrating the PerSePHONE algorithm processing steps.

The PerSePHONE (Permanent Scatterers Project Held by the Observatory, National, of Hellas) tool development, was based on the PSInSAR technique, along with a number of algorithmic adaptations for PS and PSC (Permanent Scatterer Candidate) identification and selection. Figure 3 illustrates the distinct processing steps of the algorithm elaborated in this section and used for deformation assessment in the Gulf of Corinth.

In PSInSAR technique the so called Dispersion Index (DI) is a useful indicator for the selection of the initial set of PSCs. It was obtained from the SAR amplitude images indicating a target's amplitude dispersion over time. Small values of DI indicated a possible stable target termed as PSC [2]. A modification at this stage was that the normalization of the amplitude image values, used for the calculation of the Dispersion Index (DI) and thus for the first selection of PSCs, was simplified through histogram matching between the master scene and each one of the slave scenes instead of using the calibration factor K for ERS satellites, as in [2].

The interferometric phase of each target j in each interferogram i contains the following phase components [2]:

(1) $$\varphi_{\text{i},\text{j}}=\alpha_{\text{i},\text{j}}+\varphi_{\text{topo}\_\text{res},\text{i},\text{j}}+{\text{v}}_{\text{i},\text{j}}+{\text{E}}_{\text{i},\text{j}}$$

where α contains Atmospheric Phase Screen (APS) and the orbital errors, φ_topo_res_ contains the correction for the actual target height in relation to the used DEM, v contains the deformation velocity of the radar target and E contains the effect of other components, namely non linear atmospheric disturbances, noise due to temporal and spatial decorrelation and non linear target movement.

The equation must be applied to tiles of small dimensions which will allow the estimation of the APS and the orbital errors by a 2-D linear phase approximation. Therefore the term α in each target j, in each interferogram i, in each tile t can be estimated as:

(2) $$\alpha_{\text{i},\text{j}}^{\text{t}}={\text{p}}_{\xi ,\text{i}}^{\text{t}}\xi_{\text{j}}+{\text{p}}_{\eta ,\text{i}}^{\text{t}}\eta_{\text{j}}+{\text{c}}_{\text{i}}^{\text{t}}$$

where p_ξ_, p_η_ denote the slope values along the azimuth (ξ) and the slant range (η) respectively and c denotes the constant values of the 2-D linear phase approximation.

Consequently the next step was to divide the area of interest into tiles. The test area was divided into 800 tiles each having dimensions of 500 pixels in azimuth and 100 pixels in range, covering an area of ∼4 km^2^. About 200,000 targets having DI<0.33 were identified from all tiles as a first selection of PSCs, denoted as PSCs^(1)^. Proceeding to subsequent algorithm iterations this set was reduced to PSC^(n)^, with the index n indicating the number of the iteration.

The calculation of the APS and the orbital errors (term α of equation 1) at each tile (equation 2) and a first estimate for the inherent DEM errors (term φ_topo_ of equation 1) and deformation velocities (term v of equation 1) for each PSC was performed by using a successive approximation algorithm [2, 10]. At each iteration step these values were calculated by solving a non-linear system since interferometric phase is known in modulo-2π [11]. The estimated correction quantities were then subtracted from the PSC phase values and the new interferometric phases were fed again to the same system to start the next iteration cycle. An applied modification was that in each step, the PSCs that obstructed the algorithmic convergence (either due to low DEM accuracy or due to the fact that PSC's motion could not be approximated by the constant velocity model) were removed, resulting in a new PSC^(i)^ set. The criterion for identifying the non converging PSCs was the standard deviation of the correction values at each advanced iteration step of the unknowns (velocities and DEM errors). This procedure was repeated until either the algorithm converged or the number of PSCs^(n)^ in a specific tile became lower than a minimum threshold which was set to 40. Finally, the non-linear atmospheric disturbances of low frequency were isolated from the phase residuals (term E of equation 1) by applying a kriging filter and were included in the APS estimation.

The outcome of this iterative procedure is the estimation of the APSs in 74 tiles that emerged from 4425 PSCs, having a density of ∼15 PSCs per km^2^. This value is rather limited in comparison with other research scenarios conducted in highly populated urban areas as in [2]. This happens because the study area is mainly rural and forestall, resulting in reduced signal coherence in time and therefore a lower number of PSCs was identified per Km^2^. Note though, that some tiles (especially in urban and peri-urban areas) with a larger number of initial PSCs^(n)^ were represented at the end of the iterative processing by a final density of up to 58 PSCs per km^2^ which was considered a satisfactory outcome.

The final deformation rates and precise DEM errors for each target inside a tile were then calculated. This was performed by maximizing the targets' Phase Coherence (PC), which refers to the phase stability of the targets, after removing the calculated APS from each tile [2]. This was regarded as a non-linear inverse problem, solved by means of scanning a 2-D parametric space and maximizing the PC factor emerging from the final velocities and DEM errors. During these calculations, a range of vertical displacement velocities of [-8, 8] mm year^-1^ and DEM errors of [-10, 10] m was assumed.

**Figure 4.**
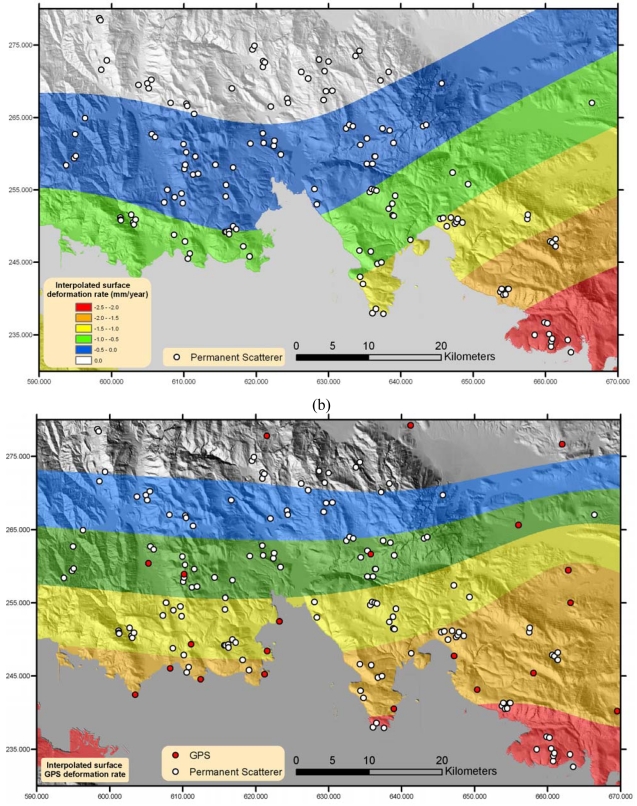
(a) Bilinear PS interpolated surface and the corresponding PSs. (b) Deformation surface derived from GPS measurements. White dots correspond to PS measurement points while red dots to GPS locations.

The last processing step was the identification of the PSs inside each tile. The Maximum PC (MPC), referring to each target, was the criterion for keeping only those that show high phase stability in time. The total number of the derived PSs was 154 and they are mapped in figure 4a. It should be noted that the above procedure incorporating interferogram calculations, PS target identification, PS processing along with the projection of the derived deformation rates to a common cartographic system, were integrated into a single processing chain named PerSePHONE, developed by Institute for Space Application and Remote Sensing of the National Observatory of Athens.

## 4. Results

Since the identified PS density per tile was rather low due to land cover incoherence and atmospheric disturbances, it was considered reasonable to continue with a surface interpolation of the observed deformation, estimating the general gradient of the velocity field in the study area, while filtering out any local anomalies. For this a thin-plate smoothing spline [12], having a smoothing parameter of p_s_=0.05, was used to best fit the returned PS velocities. As the smoothing parameter p varies from 0 to 1, the smoothing spline varies, from the least-squares approximation to the data by a linear polynomial when p is 0, to the thin-plate spline interpolant to the data when p is 1. This highlighted the general subsidence trend located in the northeast of the Gulf of Corinth study area, while the maximum deformation occurred in the region north of the Gulf of Alkyonides (∼2.5 mm year^-1^), as it became clearly visible from Figure 4a.

## 5. Validation

Marine geological and geophysical studies conducted in the Gulf of Corinth validate the above results. In [13] a mean subsidence rate of 0.7-1 m kyear^-1^ (essentially 0.7-1 mm year^-1^) of the northern margin of the Gulf of Corinth during the last 250 kyears was estimated, based on the continuous subsidence of at least four oblique prograding sequences obtained by seismic profiling in the area of Eratini. This subsidence rate is in very good agreement with the average deformation rate of ∼1 mm year^-1^ estimated from the PSInSAR study herein. Furthermore, in [14] a sequence of lowstand clinoform packages was identified in sparker profiles from the northern margin of Alkyonides basin. They have been interpreted as the subsided record of the periodic, low-frequency global sea level cycles developed over the last 0.6 Ma. In [15] the existence of these subsided clinoforms was confirmed and it was indicated that the subsidence may have initiated about 400-450 kyears ago. The above observations are also in agreement with [16], which stated that the northern margin of the gulf seems to be under regional subsidence, inferred by the sinuous shape of the coastline, the absence of any emerged fossil shorelines and the submerged ruins of Aliki (figure 1). Additionally, [13] proposed a clear northward shift of the northern coastline of the Gulf, which resulted in the formation of the shallow embayments along it, like the ones of Itea and Antikyra (figure 1). This shift was attributed solely to the regional subsidence of the northern margin of the Gulf.

An alternative approach for validating the derived PSInSAR measurements was the use of GPS height measurements collected and analyzed in the broader area of the Gulf of Corinth. These GPS campaigns were conducted by the Laboratory of Higher Geodesy of the National Technical University of Athens and the Institute of Earth Physics of Paris and lasted for approximately 13 years, starting from February 1989 till March 2002 [17]. During these campaigns more than 80 points were considered and measured at different epochs. Time series were calculated for the height component and a linear interpolation was implemented to derive the velocity vectors for each measured point. A threshold for keeping the most consistent GPS measurements was applied and this related to the square of the correlation coefficient (R^2^) of the interpolation, a measure of the reliability of the linear relationship between height measurement and time. Consequently, only points with R^2^ greater than 0.7 were kept. This simple procedure resulted in maintaining 25 points along the study area. A thin-plate smoothing spline surface was fitted to these GPS observations, using the same parametric values as for the PSInSAR surface above. A direct comparison of the two methods is presented in figures 4a and 4b. This comparison showed clearly the coincidence of both methods in the assessment of the deformation direction and location over the study area. It should be noted though, that as far as the accuracy of the vertical velocity field estimation is concerned, the PSInSAR results were more accurate and realistic than the long term GPS observations, which, as it is well known, provided vertical velocities with a higher degree of uncertainty in absolute values. Moreover, the sparse distribution of GPS points cannot lead to reliable absolute displacement values as far as the GPS interpolated surface is concerned. For this purpose a qualitative comparison between the two surfaces would be more appropriate in order to validate the deformation trend in the region than a pure comparison in absolute velocity figures. Figures 4a and 4b show that PSInSAR and GPS measurements result in a displacement pattern with the same characteristics, depicting a clear subsidence trend in the south eastern part of the northern coastline of the Gulf of Corinth.

## 6. Conclusions

In this paper the so-called PerSePHONE PSInSAR technique developed at the Institute for Space Applications and Remote Sensing of the National Observatory of Athens was implemented for systematically measuring for the first time the ground deformation velocities at the Gulf of Corinth area, over a six year period. A number of algorithmic adaptations were implemented, mainly concerning the PSC selection procedure through applying a histogram equalization technique, and the PSC iterative algorithm convergence criteria using the standard deviation of the correction values.

The Gulf of Corinth was a challenging case study for PS processing due to signal incoherence, induced by dense vegetation, high cloud coverage, frequent rainfall and lack of rocky areas and urban settlements. This analysis though, identified regions with an adequate number of PSs, providing the capability for PSInSAR processing. The stable PS targets emerged after applying strict criteria and for this reason the resulted deformation rates are considered to be accurate readings. This was confirmed by the agreement between the subsidence trend returned by the PS interpolated surface and the displacement rates reported in the relevant geophysical/geological and geodetic (GPS) studies conducted in the same area, highlighting the validity and the accuracy of the developed PSInSAR methodology.

Apart from the derived and validated deformation trend of the area, another innovative outcome of the systematic observation of the Gulf of Corinth is the identification of the exact position of potential Permanent Scatterers, information previously unpublished. This will be of paramount importance for the scientific community when selecting the location of future GPS stations, aiming at consistent observation of the region.
